# Effects of Methanol Extract of Breadfruit (*Artocarpus altilis*) on Atherogenic Indices and Redox Status of Cellular System of Hypercholesterolemic Male Rats

**DOI:** 10.1155/2014/605425

**Published:** 2014-01-30

**Authors:** Oluwatosin Adekunle Adaramoye, Olubukola Oyebimpe Akanni

**Affiliations:** Drug Metabolism and Toxicology Research Laboratories, Department of Biochemistry, College of Medicine, University of Ibadan, Ibadan 20005, Nigeria

## Abstract

We investigated the effects of methanol extract of *Artocarpus altilis* (AA) on atherogenic indices and redox status of cellular system of rats fed with dietary cholesterol while Questran (QUE) served as standard. Biochemical indices such as total cholesterol (TC), triglycerides (TG), low- and high-density lipoproteins-cholesterol (LDL-C and HDL-C), aspartate and alanine aminotransferases (AST and ALT), lactate dehydrogenase (LDH), reduced glutathione, glutathione-s-transferase, glutathione peroxidase (GPx), catalase (CAT), superoxide dismutase (SOD), and lipid peroxidation (LPO) were assessed. Hypercholesterolemic (HC) rats had significantly increased relative weight of liver and heart. Dietary cholesterol caused a significant increase (*P* < 0.05) in the levels of serum, hepatic, and cardiac TC by 110%, 70%, and 85%, LDL-C by 79%, 82%, and 176%, and TG by 68%, 96%, and 62%, respectively. Treatment with AA significantly reduced the relative weight of the organs and lipid parameters. There were beneficial increases in serum and cardiac HDL-C levels in HC rats treated with AA. In HC rats, serum LDH, ALT, and AST activities and levels of LPO were increased, whereas hepatic and cardiac SOD, CAT, and GPx were reduced. All biochemical and histological alterations were ameliorated upon treatment with AA. Extract of AA had protective effects against dietary cholesterol-induced hypercholesterolemia.

## 1. Introduction

Recent studies have demonstrated that increased formation of free radicals/reactive oxygen species (ROS) contributes to cardiovascular disease (CVD) progression [1]. Generation of large amounts of ROS can overwhelm the intracellular antioxidant defense, causing lipid peroxidation, protein modification, and DNA breaks [2]. It is known that ROS-induced depletion of antioxidants is a key factor for the initiation of atherosclerosis and the development of CVD [3]. Hypercholesterolemia, characterized by the presence of high levels of cholesterol in the blood [4], is a form of hyperlipidemia and hyperlipoproteinemia. Hyperlipidemia has also been found to induce oxidative stress in various organs of the body [5]. Although several factors, such as life style, a diet rich in cholesterol, and age, have been reported to cause heart failure [6, 7], high levels of cholesterol, particularly LDL-cholesterol, are mainly responsible for hypercholesterolemia [8]. Drugs that lower cholesterol such as fibrates and bile acid sequestrants were used for several decades, but the high prevalence of adverse effects led to the introduction of statins (HMG-CoA inhibitors) [9]. Although the adverse effect of statins is relatively low, one rare effect called rhabdomyolysis can be very serious with statins [9]. In view of these adverse effects, the quest for natural products with hypolipidemic potential and minimal side effect is warranted.

Breadfruit (*Artocarpus altilis*) is a flowering tree in the mulberry family. The fruit can be eaten once cooked or can be further processed into a variety of other foods. It is an excellent source of fiber, calcium, copper, iron, magnesium, potassium, thiamine, niacin, carbohydrates, and vitamins and very low in fat [10]. In herbal homes, leaves of this plant are used for the treatment of liver disorders, hypertension, and diabetes [11, 12]. *In vitro* studies by Nwokocha et al. [13] supported the folkloric use of this plant. However, little information is available with respect to *in vivo* studies on its ethnomedicinal uses. This study was designed to evaluate the effects of *Artocarpus altilis* on atherogenic indices and redox status of cellular system of hypercholesterolemic rats.

## 2. Materials and Methods

### 2.1. Chemicals

Questran (Bristol-Myers Squibb, Hounslow, UK) was purchased from a local chemist in Ibadan, Nigeria. Dietary cholesterol and thiobarbituric acid (TBA) were procured from Aldrich Chemical Co. (Milwaukee, WI, USA). Glutathione, hydrogen peroxide, 5,5′-dithio-bis-2-nitrobenzoic acid (DNTB), and epinephrine were purchased from Sigma Chemical Co., Saint Louis, MO, USA. Trichloroacetic acid (TCA) and thiobarbituric acid (TBA) were purchased from British Drug House (BDH) Chemical Ltd., Poole, UK. Other reagents were of analytical grade and the purest quality available.

### 2.2. Collection and Extraction of *Artocarpus altilis*

The stem bark of *Artocarpus altilis* was collected in Ibadan (Oyo State) and authenticated at the Botanical Garden of the University of Ibadan. The stem bark of *Artocarpus altilis *was air-dried and crushed into fine powder. The powdered part was extracted with n-hexane and methanol using soxhlet extractor and the extract was concentrated in vacuum at 40°C with rotary evaporator and water bath to dryness. The yield of the extraction was 5.7%.

### 2.3. Determination of Total Phenolic Contents

The total phenolic content of the extract was determined using the method of Singleton et al. [14] with slight modifications. Folin-C reagent (1 mL) was added to 1 mL of extract or standard. After 3 minutes, 1 mL of 15% Na_2_CO_3_ was added and the solution was made up to 5 mL with distilled water. The reaction mixture was kept in the dark for 90 minutes with intermittent shaking or placed in a water bath at 40°C for 20 minutes. The absorbance was measured by a Beckman DU (70) Spectrophotometer at 760 nm. All experiments were done in triplicate. A standard curve was plotted with catechin and the phenolic content expressed as CE (catechin equivalent) per mg dry weight of the extract.

#### 2.3.1. DPPH—Radical Scavenging Activity

The radical scavenging activity of the extract was measured as described by Mensor et al. [15]. The stable 2,2-diphenyl-1-picrylhydrazyl (DPPH) radical was used for the determination of free radical scavenging activities of the extracts. A portion (1 mL) of each of the different concentrations (10–1000 *μ*g/mL) of the extracts or standard (catechin) was added to 1 mL of 1 mM DPPH in methanol. The mixtures were vortexed and incubated in a dark chamber for 30 minutes after which the absorbance was measured at 517 nm against a DPPH control containing only 1 mL of methanol in place of the extract. All calculations were carried out in triplicates. The inhibition of DPPH was calculated as a percentage using the expression

(1) $$\begin{array}{c} I\%=\frac{A_{\text{control}}-A_{\text{sample}}}{A_{\text{control}}}\times 100, \end{array}$$

where *I*% is the percentage inhibition of the DPPH radical, *A*_control_ is the absorbance of the control, and *A*_sample_ is the absorbance of the test compound.

### 2.4. Animals

Inbred male Wistar rats weighing between 150 and 180 g were purchased from the animal house of the Department of Veterinary Physiology, Biochemistry, and Pharmacology, University of Ibadan, Nigeria. Animals were kept in ventilated cages at room temperature (28–30°C) and maintained on normal laboratory chow (Ladokun Feeds, Ibadan, Nigeria) and water *ad libitum*. Rats handling and treatments conform to guidelines of the National Institute of Health (NIH publication 85-23, 1985) for laboratory animal care and use. The study was approved by the Faculty of Basic Medical Sciences, University of Ibadan Animal Ethics Committee.

### 2.5. Study Design

Thirty-five male rats were randomly divided into seven groups of five rats each. The first group (control) received drug vehicle (corn oil), the second group (HC) received dietary cholesterol at 30 mg/0.3 mL [16], the third group (HC + AA1) received dietary cholesterol and *Artocarpus altilis* (100 mg/kg), the fourth group (HC + AA2) received dietary cholesterol and *Artocarpus altilis* (200 mg/kg), the fifth group (HC + QUE) received dietary cholesterol and Questran (0.26 g/kg) [16], the sixth group (QUE) received questran alone, and the seventh group (AA) received *Artocarpus altilis* at a dose of 200 mg/kg body weight.

### 2.6. Preparation of Tissues

Rats were fasted overnight and sacrificed 24 hours after the last dose of drugs. Liver and heart were quickly removed and washed in ice-cold 1.15% KCl solution, dried, and weighed. A section of liver and aorta samples were fixed in 10% formalin for histological examination. The remaining parts of liver and heart were homogenized in 4 volumes of 50 mM phosphate buffer, pH 7.4, and centrifuged at 10,000 g for 15 minutes to obtain post-mitochondrial supernatant fraction (PMF). All procedures were carried out at temperature of 0–4°C.

#### 2.6.1. Preparation of Serum

Blood was collected from the heart of the animals into plain centrifuge tubes and was allowed to stand for 1 hour. Serum was prepared by centrifugation at 3,000 g for 15 minutes in a Beckman bench centrifuge. The clear supernatant was used for the estimation of serum lipid profile and enzymes.

### 2.7. Biochemical Assays

Protein contents of the samples were assayed by the method of Lowry et al. [17] using bovine serum albumin as standard. The activities of alanine and aspartate aminotransferases (ALT and AST) were assayed by the combined methods of Mohun and Cook [18] and Reitman and Frankel [19]. Serum total cholesterol level was assayed by the method of Richmond [20]. The method involved enzymatic hydrolysis and oxidation of cholesterol with the formation of quinoneimine (an indicator) from hydrogen peroxide and 4-aminoantipyrine in the presence of phenol and peroxide. The serum level of triglyceride was determined by Jacobs and van Demark [21] and Koditschek and Umbreit [22]; this was based on the hydrolysis of triglycerides with the formation of glycerol which is substrate for other enzymes with the subsequent formation of hydrogen peroxide. This then reacts with 4-aminophenazone and 4-chlorophenol in the presence of peroxidase to give quinoneimine which is measured spectrophotometrically at 500 nm.

The lipoproteins (measured using the enzymatic colorimetric method), very low-density lipoprotein (VLDL) and low-density lipoprotein (LDL), were precipitated by the addition of phosphotungstic acid and magnesium chloride. After centrifugation at 3,000 g for 10 minutes at 25°C, the clear supernatant contained HDL fraction, which was assayed for cholesterol with the Randox diagnostic kit. The low-density lipoprotein (LDL) was calculated using the formula of Friedewald et al. [23]. Lipid peroxidation level was assayed by the reaction between 2-thiobarbituric acid (TBA) and malondialdehyde (MDA), an end product of lipid peroxides as described by Buege and Aust [24].

The activity of lactate dehydrogenase (LDH) was determined by the method of Zimmerman and Weinstein [25], while tissue superoxide dismutase (SOD) activity was measured by the nitro blue tetrazolium (NBT) reduction method of McCord and Fridovich [26]. Catalase (CAT) activity was assayed spectrophotometrically by measuring the rate of decomposition of hydrogen peroxide at 240 nm as described by Aebi [27]. Reduced glutathione level was measured by the method of Beutler et al. [28]; this method is based on the development of a relatively stable (yellow) colour when 5′,5′-dithiobis-(2-nitrobenzoic acid) (Ellman's reagent) is added to sulfhydryl compounds. The chromophoric product resulting from the reaction of Ellman's reagent with the reduced glutathione (2-nitro-5-thiobenzoic acid) possesses a molar absorption at 412 nm which is proportion to the level of reduced glutathione in the test sample. The glutathione peroxidase (GPx) activity was assessed by the method of Rotruck et al. [29], while glutathione-S-transferase (GST) activity was determined according to Habig et al. [30]; the principle is based on the fact that all of known GST demonstrates a relatively high activity with 1-chloro-2,4-dinitrobenzene as the second substrate. When this substance is conjugated with reduced glutathione, its absorption maximum shifts to a longer wavelength 340 nm and the absorption increase at this wavelength provides a direct measurement of the enzymatic reaction.

#### 2.7.1. Determination of Antiatherogenic, Cardioprotective, and Coronary Risk Indices

Cardioprotective index (CPI) was estimated in terms of HDL-C to LDL-C ratio [31, 32], whereas antiatherogenic (AAI) and coronary risk indices (CRI) were calculated by the following formulae [33, 34]:

(2) $$\begin{array}{c} \text{AAI}=100\times \frac{\left[\text{HDL-C}\right]}{\left[\text{Total}\;\text{cholesterol}-\text{HDL-C}\;\right]},\\ \text{CRI}=\frac{\text{Total}\;\text{cholesterol}}{\text{HDL-cholesterol}}. \end{array}$$

### 2.8. Histopathology of Tissues

Tissues fixed in 10% formalin were dehydrated in 95% ethanol and then cleared in xylene before embedded in paraffin. Microsections (about 4 *μ*m) were prepared and stained with haematoxylin and eosin (H&E) dye and were examined under a light microscope by a histopathologist who was ignorant of the treatment groups.

### 2.9. Statistical Analysis

All values were expressed as the mean ± SD of five animals per group. Data were analyzed using one-way ANOVA followed by the post hoc Duncan multiple range test for analysis of biochemical data using SPSS (10.0). Values were considered statistically significant at *P* < 0.05.

## 3. Results

### 3.1. Phenolic and Flavonoids Contents and Effects of *Artocarpus altilis* on Body Weight and Relative Weight of Organs of Hypercholesterolemic (HC) Rats

In Table 1, there were significant increases (*P* < 0.05) in the relative weight of liver and heart of HC rats when compared with the control, while treatment with AA (100 and 200 mg/kg) significantly reduced the relative weight of heart and liver of HC rats to values that were statistically similar (*P* > 0.05) to the control. Similar reduction was obtained in questran-treated HC rats. The total phenolic contents (TPC) of AA expressed in *μ*g catechin equivalent per mg dry weight of the extract increased with increase in concentration (Table 2). At 750 *μ*g/mL, the TPC of AA was 0.68 ± 0.05 *μ*g CE/mg. There were significant (*P* < 0.05) and dose-dependent increases in scavenging activity of AA on DPPH radicals (Table 2). At 100 *μ*g/mL and 750 *μ*g/mL, the percentage DPPH radical scavenging activities of AA were 42.2% and 67.8%, respectively.

### 3.2. Effects of *Artocarpus altilis* on Antioxidant Parameters and Marker Enzymes in Hypercholesterolemic (HC) Rats

Administration of dietary cholesterol significantly increased (*P* < 0.05) serum, hepatic, and cardiac lipid peroxidation (LPO) products measured as thiobarbituric acid reactive substances (TBARS) by 265%, 83%, and 80%, respectively (Table 3). However, treatment with AA completely ameliorated dietary cholesterol-induced increase in LPO. In HC rats, the activities of hepatic, and cardiac SOD and CAT as well as cardiac GPx decreased significantly relative to the control (Table 4). Specifically, hepatic SOD and CAT decreased by 54% and 45%, while cardiac SOD, CAT, and GPx decreased by 67%, 59%, and 36%, respectively. Also, activities of phase II and antioxidant enzyme (GST) in the liver of HC rats were significantly reduced when compared to controls (Figure 5). Administration of AA (200 mg/kg) reversed the adverse effect of high dietary cholesterol by normalizing these enzymic antioxidant indices. In HC rats, serum ALT, AST and LDH were significantly increased by 2.3-, 1.7-, and 2.4-fold, respectively, while cardiac LDH activity was decreased by 3.0-fold relative to controls (Table 5 and Figures 3 and 4). However, the observed elevations in the activities of these serum enzymes in HC rats were reversed following treatment with AA and quetsran.

### 3.3. Effects of *Artocarpus altilis* on the Lipid Profile of Hypercholesterolemic Rats

**Figure 1:**
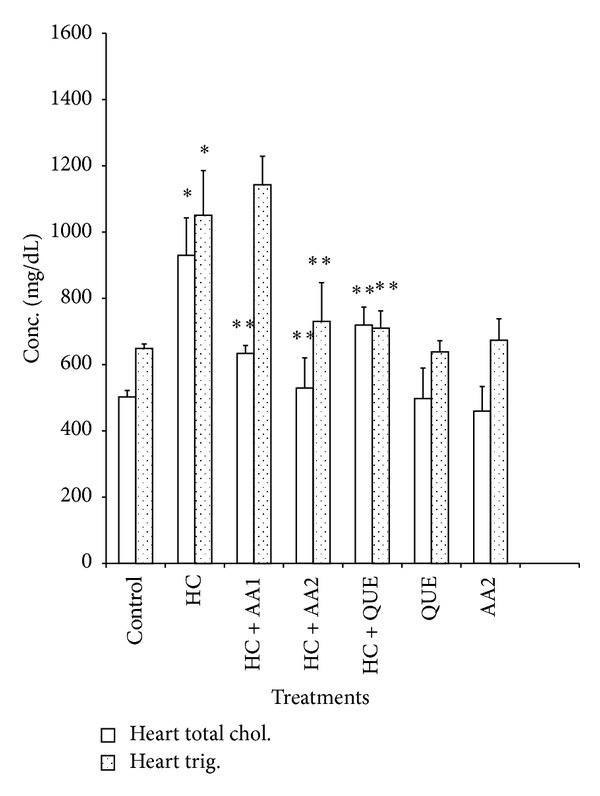
Effects of methanol extract of *Artocarpus altilis* and Questran on cardiac total cholesterol and triglyceride levels of hypercholesterolemic rats. *Significantly different from control (*P* < 0.05), **significantly different from HC (*P* < 0.05). HC: Hypercholesterolemic rats, AA1: *Artocarpus altilis* at 100 mg/kg, AA2: *Artocarpus altilis* at 200 mg/kg, and QUE: Questran at 0.26 g/kg.

**Figure 2:**
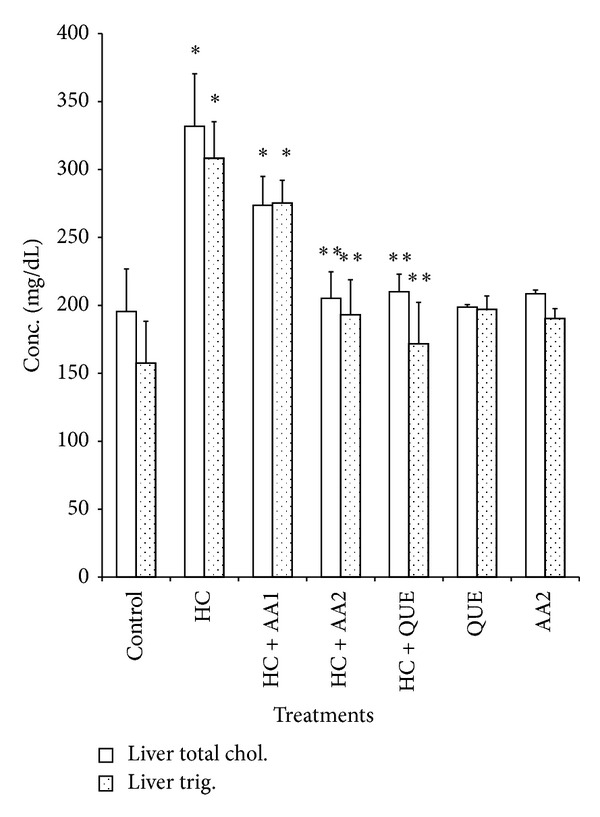
Effects of methanol extract of *Artocarpus artilis* and Questran on hepatic total cholesterol and triglyceride levels of hypercholesterolemic rats. *Significantly different from control (*P* < 0.05), **significantly different from HC (*P* < 0.05). HC: hypercholesterolemic rats, AA1: *Artocarpus altilis* at 100 mg/kg, AA2: *Artocarpus altilis* at 200 mg/kg, and QUE: Questran at 0.26 g/kg.

**Figure 3:**
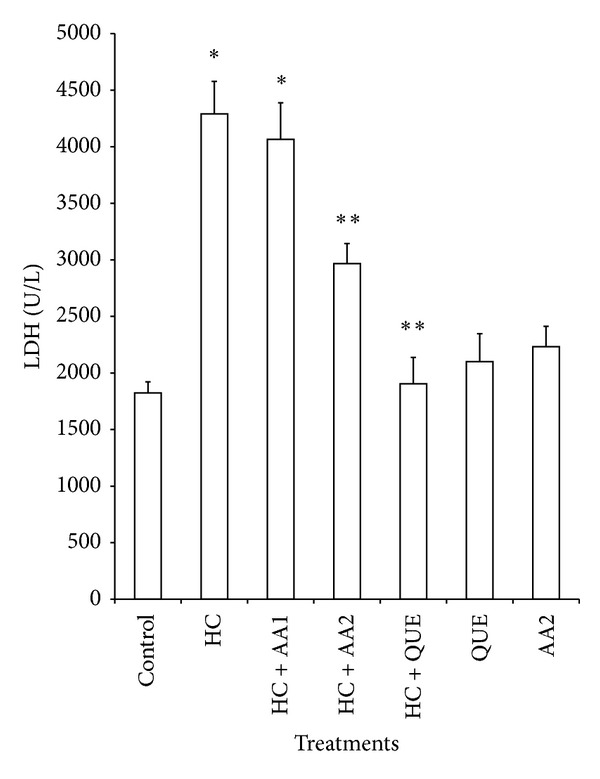
Effects of methanol extract of *Artocarpus artilis* on the activities of serum lactate dehydrogenase (LDH) of hypercholesterolemic rats. *Significantly different from control (*P* < 0.05), **significantly different from HC (*P* < 0.05). HC: hypercholesterolemic rats, AA1: *Artocarpus altilis* at 100 mg/kg, AA2: *Artocarpus altilis* at 200 mg/kg, and QUE: Questran at 0.26 g/kg.

**Figure 4:**
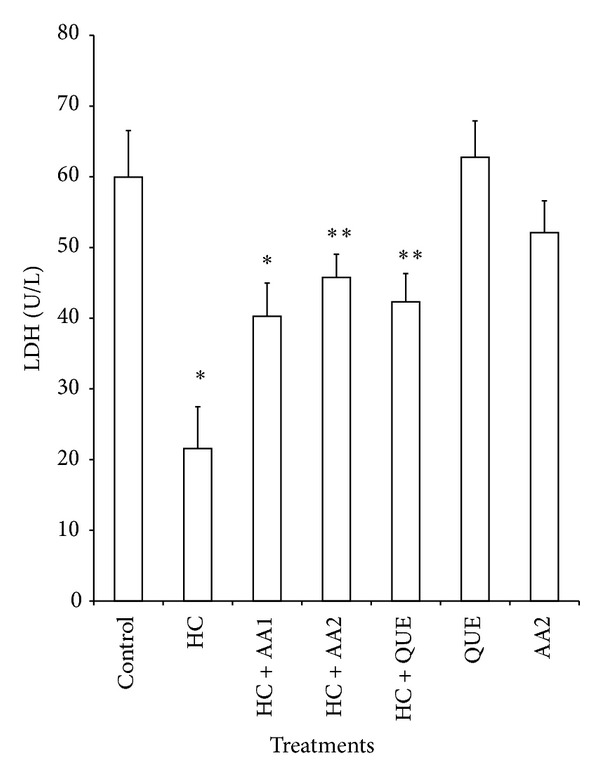
Effects of methanol extract of *Artocarpus artilis* on the activities of cardiac lactate dehydrogenase (LDH) of hypercholesterolemic rats. *Significantly different from control (*P* < 0.05), **significantly different from HC (*P* < 0.05). HC: hypercholesterolemic rats, AA1: *Artocarpus altilis* at 100 mg/kg, AA2: *Artocarpus altilis* at 200 mg/kg, and QUE: Questran at 0.26 g/kg.

**Figure 5:**
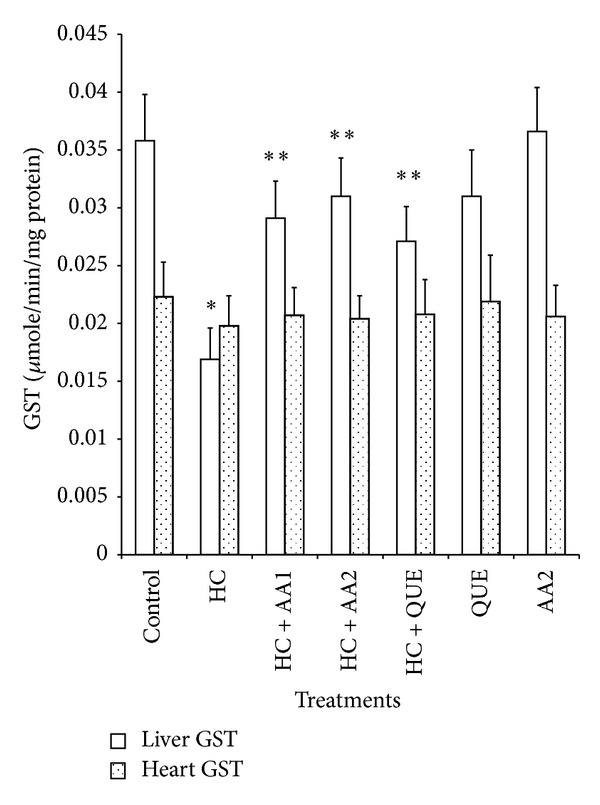
Effects of methanol extract of *Artocarpus artilis *on the activities of hepatic and cardiac glutathione-s-transferase (GST) of hypercholesterolemic rats. *Significantly different from control (*P* < 0.05), **significantly different from HC (*P* < 0.05). HC: hypercholesterolemic rats, AA1: *Artocarpus altilis* at 100 mg/kg, AA2: *Artocarpus altilis* at 200 mg/kg, and QUE: Questran at 0.26 g/kg.

Feeding rats on high dietary cholesterol for nine consecutive weeks significantly (*P* < 0.05) increased the serum, hepatic, and cardiac total cholesterol levels by 110%, 70%, and 85%, respectively (Table 6 and Figures 1 and 2). Furthermore, serum, hepatic, and cardiac triglycerides increased by 68%, 96%, and 62%, while serum LDL-C increased by 79%, respectively, in HC rats relative to controls. In addition, HC rats had significantly lower HDL-C values when compared to the control (Table 6). Administration of AA at 200 mg/kg attenuated the elevated levels of these lipid indices to near normal in the tissues of HC rats. The protective effect of AA at 200 mg/kg seems better than the standard hypolipidemic drug (Questran). Furthermore, AA increased serum antiatherogenic index in HC rats, while coronary risk index was decreased (Table 7).

### 3.4. Effects of *Artocarpus altilis* on the Histology of Aorta and Liver

**Figure 6:**
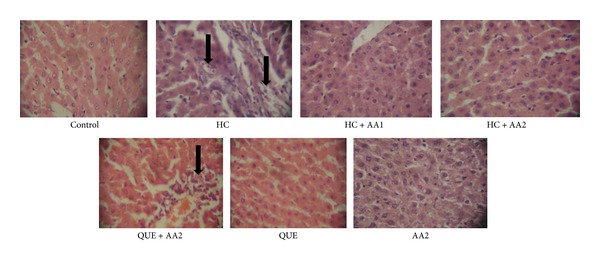
Changes in histology of liver samples of hypercholesterolemic rats treated with *Artocarpus altilis* and Questran for nine consecutive weeks (M ×400). HC: cholesterol at 30 mg/0.3 mL, AA1: *Artocarpus altilis* at 100 mg/kg, AA2: *Artocarpus altilis* at 200 mg/kg, and QUE: Questran at 0.26 g/kg. Black arrow shows portal congestion, periportal cellular infiltration, and vacuolar degeneration of hepatocytes.

**Figure 7:**
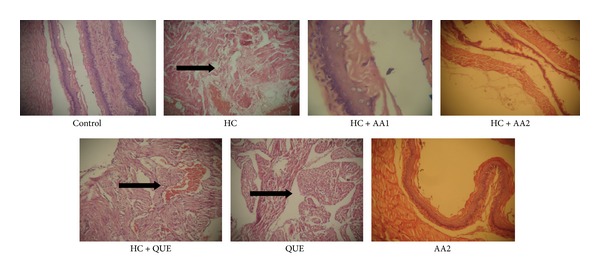
Changes in histology of aorta from hypercholesterolemic rats treated with *Artocarpus altilis* and Questran for nine consecutive weeks (M ×400). HC: cholesterol at 30 mg/0.3 mL, AA1: *Artocarpus altilis* at 100 mg/kg, AA2: *Artocarpus altilis* at 200 mg/kg, and QUE: Questran at 0.26 g/kg. Black arrows shows myofibril necrosis, severe hemorrhages, and fibrous connective tissue laid down.

The histology of liver slide showed marked portal congestion, severe periportal cellular infiltration by mononuclear cells, and mild diffuse vacuolar degeneration of hepatocytes (Figure 6), while aorta from HC rats revealed large focal area of myofibril necrosis with severe hemorrhages and fibrous connective tissue laid down (Figure 7). Treatment with AA (200 mg/kg) reversed the adverse effect of high dietary cholesterol on the histological architecture of the aorta and liver of the rats. The histological results further corroborated the biochemical findings indicating the beneficial effects of AA in hypercholesterolemic rats.

## 4. Discussion

It is generally known that elevation of serum LDL-C and total cholesterol (TC) can lead to CVD, especially atherosclerosis. Reducing LDL-C and TC can prevent the risk of CVD, a leading cause of mortality worldwide [35]. Appropriate lifestyle changes and pharmacologic approaches have both demonstrated their effectiveness in lowering LDL-C and TC [36], but the negative side effects of the pharmacological intervention have been a major setback. Lifestyle changes to include decreased saturated fats and increased soluble fibre in the diet, weight loss and regular physical activity are primary strategy for preventing CVD. Regular consumption of dietary supplements or functional foods that have demonstrated positive effects on plasma lipid values in randomised placebo-controlled clinical studies can also be considered as part of this CVD prevention strategy [37]. On this basis, we investigated the effects of methanol extract of AA on atherogenic indices and redox status of cellular system in hypercholesterolemic (HC) rats.

The present study clearly shows that feeding rats on high cholesterol diets for nine weeks caused significant increase in relative weight of heart and liver of the rats. This observation is consistent with the findings of Adaramoye et al. [16] and Yuji et al. [38]. However, treatment with AA (100 and 200 mg/kg) and Questran significantly reduced the relative weight of liver and heart of the HC rats. In this study, HC rats had high serum, hepatic, and cardiac TC, TG, and LDL-C when compared to controls. Similar observations on hypercholesterolemic rats were observed by Yuji et al. [38], Adaramoye et al. [39], and, Kamesh and Sumathi [40]. Furthermore, a decrease in serum HDL-C levels was also observed in HC rats, which actually reflects the lower cholesterol transports by HDL-C in blood from peripheral tissues to liver for its metabolism and excretion [41]. The elevated serum and tissues levels of TC, TG, and LDL-C and lower levels of HDL-C provide a high risk for the development of atherosclerosis and other CVD [42]. In the study, extract of AA significantly decreased the levels of TC, TG, and LDL-C and increased HDL-C in the HC rats as compared to controls. The lipoproteins, especially LDL-C are involved in depositing TC and TG on walls of coronary arteries and initiate the process of atherosclerotic plaques [43]. Reduced serum and tissues levels of TC, TG, and LDL-c found in HC rats treated with doses (100 and 200 mg/kg) of AA are among the beneficial aspects of this current research and proved the antiatherosclerotic potential of this extract. The crucial risk factor for CVD includes a low level of HDL-C and high level of LDL-C. The association between a low level of HDL-C and an increased risk of CVD has been well established through epidemiological and clinical studies [44]. Since low level of HDL-C plays a direct role in the atherogenic process, therapeutic intervention to raise HDL-C together with other risk factors is widely encouraged. In this study, treatment with AA led to significant elevation of HDL-C, indicating its promising protective role against CVD. The protective roles of HDL-C from CVD have been suggested to occur in various ways [45]. HDL exerts part of its antiatherogenic effect by counteracting LDL oxidation and studies also showed that HDL promotes the reverse cholesterol transport pathway, by inducing an efflux of excess accumulated cellular cholesterol, and prevents the generation of an oxidatively modified LDL [46]. Furthermore, HDL not only inhibits the oxidation of LDL by transition metal ions but also prevents 12-lipoxygenase-mediated formation of lipid hydroperoxides [45]. On the basis of our results, AA may probably plays an antiatherogenic role through the inhibition of lipids oxidation, due to its antilipoperoxidative effect observed in this study as well as the elevation of HDL-C. LDL-C, another primary target of CVD risk reduction therapy [41]. In this study, AA administered at a dose of 200 mg/kg lowered LDL-C levels of hypercholesterolemic rats. It is known that excess of LDL can be deposited on the blood vessel walls and becomes a major component of atherosclerotic plaque lesions. Therefore, serum LDL-C level has been used to monitor treatment of patients with elevated blood cholesterol levels. In view of our results, AA elicited beneficial effects by lowering serum total cholesterol including low-density lipoprotein of the hypercholesterolemic rats. In addition, hypocholesterolemic and hypotriglyceridemic effects of AA may probably be due to the inhibition of rate-limiting enzyme 3-hydroxy-3-methyl glutaryl CoA reductase (HMG-CoA reductase) of cholesterol biosynthesis. The experimentally obtained hypotriglyceridemic effect of AA may also be due to the improvement in lipolysis by reducing the activity of hormone-sensitive lipase [42]. To further support the lipid lowering potential of AA, the antiatherogenic index (AAI) was also evaluated and found to increase in HC rats treated with AA as compared to controls. Similarly, improvement was also observed in cardioprotective index (CPI) of HC rats treated with AA in terms of HDL-C/LDL-C ratio relative to controls. Out of the risk indices considered, HDL-C/LDL-C ratio (CPI) was found ideal in the present study. It has been reported that a decrease in HDL-C/LDL-C ratio is good predictor of CVD in subjects [47]. Similarly, coronary risk index (CRI) in terms of TC/HDL-C ratio significantly decreased in AA-treated HC rats, which further strengthen the beneficial effects of AA.

Serum AST and ALT are the reliable markers for liver function, while serum LDH may give information on the state of the cardiac tissue. It is established that AST can be found in the liver, cardiac muscle, skeletal muscle, and so forth, whereas ALT is predominantly present in the liver [48]. The increased levels of serum AST and ALT in HC rats indicate an increased permeability and damage and/or necrosis of hepatocytes. Similar results were reported by Suk et al. [49] and Mohd Esa et al. [50] in which ALT and AST activities were elevated in HC rats. In our study, we found that extract of AA at a dose of 200 mg/kg caused a significant reduction in the activities of serum AST, ALT, and LDH, which further supports the beneficial effects of the extract of AA in HC rats.

Oxidative stress, defined as a disruption of the balance between oxidative and antioxidative processes, plays an important role in the pathogenesis of atherosclerosis [51]. Studies in animal models and human clinical trials have established a relationship between hypercholesterolemia and lipid peroxidation [50]. In agreement with these findings, our results show increased levels of MDA in the serum and tissues of HC rats when compared to controls. On the other hand, treatment with AA caused a significant reduction in the levels of MDA in these organs. This protective effect is probably based on the antioxidant activity of AA, which reduced the oxidative damage by blocking the production of free radicals and thus inhibited lipid peroxidation. In this study, we also observed a significant decrease in the activities of free radical scavenging enzymes, SOD and CAT, which are the first line of defence against oxidative injury. The inhibition of antioxidant system (SOD and CAT) may cause the accumulation of H_2_O_2_ or products of its decomposition [52]. SOD catalyzes the conversion of superoxide anion into H_2_O_2_. The primary role of CAT is to scavenge H_2_O_2_ that has been generated by free radical or by SOD. Importantly, administration of AA restored the activities of enzymatic antioxidants (SOD and CAT) in liver and heart of HC rats. AA may therefore act as an effective antioxidant of great importance against diseases and degenerative processes caused by oxidative stress. Our results showed that the extract of AA at 750 *μ*g/mL produced 63% inhibition of DPPH radical relative to catechin (68%). The antioxidant property of AA may be linked to high polyphenolic compounds in this plant as shown in our results. From these findings, AA positively modulates the antioxidant redox status of HC rats, in addition to its beneficial effects on the lipid profile.

## 5. Conclusion

The present study suggests that *Artocarpus altilis* has potent blood and tissues lipid-lowering capability. In addition, it has significant antiatherogenic effect and also improves antioxidant system of hypercholesterolemic rats. Further studies are required to identify the active component(s) and mechanism(s) underlying the beneficial effects of this plant.

## Figures and Tables

**Table 1:** Changes in the body weight and relative weight of organs of hypercholesterolemic rats treated with methanol extract of *Artocarpus  altilis* for nine weeks.

Treatment	Body weight (g)		Weight of organs (g)			Relative weight of organs		
	Initial	Final	Liver	Kidney	Heart	Liver	Kidney	Heart
Control	151.00 ± 4.94	194.00 ± 23.02	5.38 ± 0.49	1.03 ± 0.11	0.53 ± 0.09	2.77 ± 0.75	0.53 ± 0.05	0.27 ± 0.04
HC	160.00 ± 3.10	198.00 ± 29.03	6.89 ± 0.89	1.09 ± 0.14	0.69 ± 0.06	3.48 ± 0.23*	0.55 ± 0.03	0.39 ± 0.02*
HC + AA1	159.00 ± 5.48	196.00 ± 37.15	5.12 ± 1.22	1.09 ± 0.20	0.55 ± 0.08	2.61 ± 0.13**	0.56 ± 0.04	0.28 ± 0.03**
HC + AA2	167.00 ± 6.04	185.00 ± 45.00	5.20 ± 1.24	1.09 ± 0.20	0.52 ± 0.09	2.81 ± 0.15**	0.59 ± 0.06	0.28 ± 0.04**
HC + QUE	173.00 ± 4.74	202.50 ± 5.00	5.39 ± 1.05	1.19 ± 0.07	0.55 ± 0.01	2.66 ± 0.43**	0.59 ± 0.05	0.27 ± 0.01**
QUE	188.00 ± 3.95	225.00 ± 20.41	5.06 ± 1.21	1.17 ± 0.05	0.51 ± 0.49	2.24 ± 0.41	0.52 ± 0.07	0.23 ± 0.04
AA2	192.00 ± 8.34	235.00 ± 22.36	5.19 ± 0.55	1.21 ± 0.11	0.62 ± 0.06	2.21 ± 0.17	0.52 ± 0.04	0.26 ± 0.03

Values are means ± SD of 5 animals per group; HC: cholesterol at 30 mg/0.3 mL.

AA1: *Artocarpus  altilis* at 100 mg/kg, AA2: *Artocarpus  altilis* at 200 mg/kg, and QUE: Questran at 0.26 g/kg.

*Significantly different from control (*P* < 0.05), **significantly different from HC (*P* < 0.05).

**Table 2:** The total phenolic contents and scavenging activity of *Artocarpus  altilis* on 2,2-diphenyl-1-picrylhydrazyl radical (DPPH) *in  vitro*.

Conc.	% Scavenging activity		Phenolic content
(*μ*g/mL)	Catechin	AA	(*μ*g CE/mg)
100	42.2 ± 4.4	22.7 ± 5.9	0.18 ± 0.02
300	47.6 ± 1.6	50.4 ± 3.1	0.39 ± 0.03
500	63.1 ± 5.1	51.1 ± 2.3	0.52 ± 0.07
750	67.8 ± 3.9	62.5 ± 8.5	0.68 ± 0.05

Data are expressed as mean ± SD (*n* = 4).

AA: *Artocarpus  altilis*.

**Table 3:** Changes in the levels of lipid peroxidation in hypercholesterolemic rats treated with methanol extract of *Artocarpus  altilis* for nine weeks.

Treatments	Liver (*μ*mol MDA/mg protein)	Heart (*μ*mol MDA/mg protein)	Serum (*μ*mol MDA/mg protein)
Control	0.06 ± 0.01	0.15 ± 0.02	1.32 ± 0.24
HC	0.11 ± 0.02*	0.27 ± 0.03*	4.82 ± 0.68*
HC + AA1	0.08 ± 0.03**	0.13 ± 0.03**	2.63 ± 0.56**
HC + AA2	0.07 ± 0.02**	0.14 ± 0.03**	2.04 ± 0.35**
HC + QUE	0.08 ± 0.02	0.15 ± 0.02	2.85 ± 0.77
QUE	0.05 ± 0.01	0.16 ± 0.07	1.82 ± 0.60
AA2	0.07 ± 0.01	0.15 ± 0.02	1.16 ± 0.33

Values are means ± SD of 5 animals per group; HC: cholesterol at 30 mg/0.3 mL.

AA1: *Artocarpus  altilis* at 100 mg/kg, AA2: *Artocarpus  altilis* at 200 mg/kg, and QUE: Questran at 0.26 g/kg.

*Significantly different from control (*P* < 0.05), **significantly different from HC (*P* < 0.05).

**Table 4:** Changes in the levels of hepatic and cardiac antioxidant parameters in hypercholesterolemic rats treated with methanol extract of *Artocarpus  altilis* for nine weeks.

Treatment	Liver				Heart			
	GSH	GPx	SOD	CAT	GSH	GPx	SOD	CAT
	(mg/g tissue)		(U/mg protein)		(mg/g tissue)		(U/mg protein)	
Control	0.85 ± 0.15	5.55 ± 0.83	7.36 ± 1.01	5.57 ± 1.08	21.06 ± 1.11	145.74 ± 5.74	0.03 ± 0.01	5.37 ± 0.81
HC	0.73 ± 0.01	4.92 ± 0.08	3.40 ± 0.69*	3.04 ± 0.05*	19.25 ± 0.99	92.82 ± 2.87*	0.01 ± 0.01*	2.19 ± 0.65*
HC + AA1	0.92 ± 0.26	4.98 ± 0.06	7.26 ± 1.00**	5.43 ± 1.71**	19.35 ± 0.85	108.43 ± 2.87	0.01 ± 0.01	2.99 ± 0.74
HC + AA2	0.96 ± 0.25	5.73 ± 1.50	7.43 ± 0.71**	5.55 ± 1.59**	20.38 ± 1.11	137.47 ± 3.01**	0.03 ± 0.00**	4.03 ± 0.80**
HC + QUE	0.85 ± 0.12	5.37 ± 0.99	5.95 ± 0.24	5.32 ± 3.90	21.21 ± 3.10	147.50 ± 4.81	0.03 ± 0.01	5.65 ± 0.74
QUE	0.79 ± 0.08	4.55 ± 0.69	5.39 ± 0.86	4.90 ± 0.59	20.24 ± 1.66	130.64 ± 3.39	0.02 ± 0.00	4.55 ± 0.90
AA2	0.88 ± 0.04	5.24 ± 0.12	6.03 ± 0.56	5.11 ± 0.04	20.46 ± 0.77	144.46 ± 3.83	0.03 ± 0.01	3.92 ± 0.91

Values are means ± SD of 5 animals per group; HC: cholesterol at 30 mg/0.3 mL.

AA1: *Artocarpus  altilis* at 100 mg/kg, AA2: *Artocarpus  altilis* at 200 mg/kg, and QUE: Questran at 0.26 g/kg.

*Significantly different from control (*P* < 0.05), **significantly different from HC (*P* < 0.05).

**Table 5:** Changes in the activities of serum, hepatic, and cardiac alanine and aspartate aminotransferases in hypercholesterolemic rats treated with methanol extract of *Artocarpus  altilis* for nine weeks.

Treatments	Liver (U/L)		Heart (U/L)		Serum (U/L)	
	AST	ALT	AST	ALT	AST	ALT
Control	610.6 ± 16.3	70.2 ± 5.4	552.2 ± 24.2	317.3 ± 12.3	218.0 ± 18.0	52.0 ± 7.6
HC	662.5 ± 19.3	72.7 ± 9.5	586.8 ± 21.2	330.2 ± 19.5	362.8 ± 11.0*	121.8 ± 10.5*
HC + AA1	642.8 ± 13.7	69.4 ± 8.8	549.4 ± 17.8	341.0 ± 15.0	243.8 ± 17.0**	73.3 ± 4.3**
HC + AA2	641.3 ± 19.5	67.93 ± 7.2	571.2 ± 25.3	309.4 ± 15.6	258.7 ± 13.0**	70.8 ± 4.8**
HC + QUE	642.2 ± 15.0	66.9 ± 01.8	558.7 ± 18.2	282.±24.8	263.5 ± 11.0**	63.5 ± 8.5
QUE	617.5 ± 15.3	69.3 ± 10.5	569.1 ± 13.54	274.0 ± 11.3	211.0 ± 9.6	60.4 ± 7.6
AA2	617.0 ± 10.6	67.0 ± 7.9	533.0 ± 22.5	312.2 ± 17.4	229.4 ± 16.5	64.0 ± 6.6

Values are means ± SD of 5 animals per group; HC: cholesterol at 30 mg/0.3 mL.

AA1: *Artocarpus  altilis* at 100 mg/kg, AA2: *Artocarpus  altilis* at 200 mg/kg, and QUE: Questran at 0.26 g/kg.

*Significantly different from control (*P* < 0.05), **significantly different from HC (*P* < 0.05).

**Table 6:** Changes in serum lipid profile of hypercholesterolemic rats treated with methanol extract of *Artocarpus  altilis* for nine weeks.

Treatment	Total chol.	Triglyceride (mg/dL)	LDL-C	HDL-C
Control	325.07 ± 7.71	416.51 ± 24.92	211.40 ± 9.03	242.13 ± 29.55
HC	680.81 ± 16.42*	701.77 ± 18.63*	376.55 ± 13.71*	150.39 ± 32.49*
HC + AA1	511.19 ± 13.99**	521.44 ± 15.13**	366.04 ± 14.21	110.15 ± 14.00
HC + AA2	464.07 ± 16.65**	484.88 ± 11.03**	257.39 ± 16.50**	228.52 ± 16.67**
HC + QUE	378.71 ± 12.59	452.46 ± 13.17	308.28 ± 10.95	223.01 ± 14.67
QUE	411.33 ± 16.72	409.51 ± 14.08	298.72 ± 16.19	176.19 ± 51.72
AA2	345.54 ± 11.01	438.69 ± 18.63	228.18 ± 10.21	242.78 ± 17.18

Values are means ± SD of 5 animals per group; HC: cholesterol at 30 mg/0.3 mL.

AA1: *Artocarpus  altilis* at 100 mg/kg, AA2: *Artocarpus  altilis* at 200 mg/kg, and QUE: Questran at 0.26 g/kg,

*Significantly different from control (*P* < 0.05).

**Significantly different from HC (*P* < 0.05).

**Table 7:** Changes in antiatherogenic, coronary risk, and cardioprotective indices of hypercholesterolemic rats treated with methanol extract of *Artocarpus  altilis* and Questran for nine weeks.

Treatment	Serum		
	AAI (%)	CRI	CPI
Control	292.1 ± 21.2	1.34 ± 0.03	1.15 ± 0.05
HC	28.0 ± 3.7*	4.53 ± 0.25*	0.40 ± 0.06*
HC + AA1	27.2 ± 2.7*	4.65 ± 0.43*	0.30 ± 0.05*
HC + AA2	116.1 ± 17.3**	1.86 ± 0.17**	0.89 ± 0.15**
HC + QUE	143.0 ± 21.8**	1.70 ± 0.22**	0.72 ± 0.08**
QUE	149.1 ± 12.1	1.67 ± 0.26	0.83 ± 0.10
AA2	233.0 ± 22.7	1.43 ± 0.22	1.06 ± 0.41

HC: cholesterol at 30 mg/0.3 mL, AA1: *Artocarpus  altilis* at 100 mg/kg,

AA2: *Artocarpus  altilis* at 200 mg/kg, QUE: questran at 0.26 g/kg,

antiatherogenic index (AAI): 100 × [HDL-C/total cholesterol−HDL-C],

coronary risk index (CRI): total cholesterol/HDL-C,

cardioprotective index (CPI): HDL-C/LDL-C.

*Significantly different from control (*P* < 0.05), **significantly different from HC (*P* < 0.05).
